# Serological analysis of allergic components of house dust mite provides more insight in epidemiological characteristics and clinical symptom development in North China

**DOI:** 10.3389/fimmu.2023.1083755

**Published:** 2023-04-27

**Authors:** Yi Liu, Lan Zhao, Jiaofeng Wang, Yinshi Guo, Yifei Wang, Lishan Zhang, Zhoujie Wu, Mingzhi Zhu, Xukai Yang, Puyang Xu, Shandong Wu, Zhongshan Gao, Jin-Lyu Sun

**Affiliations:** ^1^ Allergy Department, State Key Laboratory of Complex Severe and Rare Diseases, Peking Union Medical College Hospital, Chinese Academy of Medical Sciences and Peking Union Medical College, Beijing, China; ^2^ R&D Department, Hangzhou Zheda Dixun Biological Gene Engineering Co. Ltd., Zhejiang, China; ^3^ College of Agriculture and Biotechnology, Zhejiang University, Hangzhou, China; ^4^ Institute of Immunology, School of Medicine, Zhejiang University, Hangzhou, China; ^5^ Allergy Department, Renji Hospital Affiliated to Shanghai Jiaotong University School of Medicine, Shanghai, China

**Keywords:** allergen component, house dust mite allergy, serological analysis, IgE, clinical epidemiology

## Abstract

**Background:**

House dust mite (HDM) is the most common airborne source causing complex allergy symptoms. There are geographic differences in the allergen molecule sensitization profiles. Serological testing with allergen components may provide more clues for diagnosis and clinical management.

**Objective:**

This study aims to investigate the sensitization profile of eight HDM allergen components in a large number of patients enrolled in the clinic and to analyze the relation of gender, age, and clinical symptoms in North China.

**Methods:**

The 548 serum samples of HDM-allergic patients (ImmunoCAP^®^ d1 or d2 IgE ≥0.35) were collected in Beijing City and divided in four different age groups and three allergic symptoms. The specific IgE of HDM allergenic components, Der p 1/Der f 1, Der p 2/Der f 2, Der p 7, Der p 10, Der p 21, and Der p 23, was measured using the micro-arrayed allergen test kit developed by Hangzhou Zheda Dixun Biological Gene Engineering Co., Ltd. The new system was validated by comparing to single-component Der p 1, Der p 2, and Der p 23 tests by ImmunoCAP in 39 sera. The epidemiological study of these IgE profiles and the relation to age and clinical phenotypes were analyzed.

**Results:**

A greater proportion of male patients was in the younger age groups, while more female patients were in the adult groups. Both the sIgE levels and the positive rates (approximately 60%) against Der p 1/Der f 1 and Der p 2/Der f 2 were higher than for the Der p 7, Der p 10, and Der p 21 components (below 25%). The Der f 1 and Der p 2 positive rates were higher in 2–12-year-old children. The Der p 2 and Der f 2 IgE levels and positive rates were higher in the allergic rhinitis group. The positive rates of Der p 10 increased significantly with age. Der p 21 is relevant in allergic dermatitis symptom, while Der p 23 contributes to asthma development.

**Conclusion:**

HDM groups 1 and 2 were the major sensitizing allergens, with group 2 being the most important component relevant to respiratory symptoms in North China. The Der p 10 sensitization tends to increase with age. Der p 21 and Der p 23 might be associated with the development of allergic skin disease and asthma, respectively. Multiple allergen sensitizations increased the risk of allergic asthma.

## 1 Introduction

An allergic disease seriously affects the quality of life of patients. High medical costs and social and economic impact have made it a major global public health problem, especially in fast-developing countries like China (1, 2). House dust mite (HDM), the most common source of indoor allergens, induces a variety of allergic diseases, including allergic rhinitis, conjunctivitis, allergic asthma, and atopic dermatitis. HDM is the major cause of allergy in warm and humid areas. The dominant species of HDMs in most areas are *Dermatophagoides pteronyssinus* and *Dermatophagoides farinae*, with different proportions depending on the geographic location (3).

More than 30 HDM molecular allergens have been identified from *D. pteronyssinus* and *D. farinae*, of which groups 1, 2, 5, 7, 21, and 23 are clinically the most relevant according to the frequency of IgE sensitization and the ability to induce allergic reactions (4, 5). Der p 10 is evolutionarily conserved: it cross-reacts among organisms such as shellfish and parasites (6, 7) and is known to be an important inducer of severe systemic anaphylaxis (8). Significant variations reported in the incidence of IgE-mediated allergy, triggered by major and relevant minor mite allergens, may be caused by differences in geographic areas (9), age, and clinical phenotypes of the study populations (10). There are relatively few clinical studies of HDM components related to China, the biggest developing country with a substantial population and vast territory (11–15). The sensitization profiles for HDM component allergens differ considerably among distinct geographic regions and allergic diseases (12). In China, especially South China, Der p 1 and group 2 (Der p 2/Der f 2) have been shown to be the dominant allergens in patients with HDM allergy (12, 15), with the relative importance of Der p 2 being higher (15). South China has also been found to have the highest sensitization and levels of HDM allergens (12). Allergic rhinitis (AR) patients showed more frequent sensitization to Der p 1 and Der p 2 than asymptomatic subjects sensitized to HDM (14).

Nowadays, the immunotherapy of HDM-allergic diseases still relies on the preparation of allergen extracts. *D. pteronyssinus* and *D. farinae* extracts have also been used for allergen immunotherapy (AIT) treatment generally (16). However, there is a problem of large individual differences in the effects of treatment because the sensitization profiles of main allergen molecules and variable allergen extracts contain a variety of true allergenic and non-allergenic components (17). With the difficulty in standardizing raw materials and processing methods, allergen extracts have been shown to vary in allergen content, which can affect the accuracy of the test results. In recent years, the research of allergens has gradually been refined and developed at the molecular level, and now component-resolved diagnostics (CRD), proposed by Rudolf Valenta et al. (17), can identify the specific molecular allergen (18). This approach can help explain the potential molecular basis of cross-reactions (11), evaluate the risk of allergic reactions, identify the main molecules responsible for symptoms, and identify patients suitable for specific immunotherapy (10, 19). According to recent publications on mite CRD, Der p 1, Der p 2, Der p 7, Der p 10, Der p 21, Der p 23, Der f 1, and Der f 2 are the most relevant allergens in HDM-allergic patients (20–25).

In China, multiple allergens have been used in clinical studies on *Artemisia* pollen allergy (26) by ImmunoCAP and milk allergy with the microarrayed allergen test kit developed by Dixun Company in China (27), but there is a lack of comprehensive research with major and minor mite allergen components.

In our study, 548 HDM-allergic patients were diagnosed with three main symptoms—allergic asthma (asthma), AR, allergic skin disease (AD)—and their serum samples were analyzed with a panel of specific IgE to Der p 1, Der p 2, Der p 7, Der p 10, Der p 21, Der p 23, Der f 1, and Der f 2. The possible associations of the symptoms with age, gender, and clinical phenotypes were evaluated to obtain valuable information to assist treatment and scientific research.

## 2 Materials and methods

### 2.1 Patients and symptoms

A total of 548 serum samples from patients with confirmed dust mite allergy, identified by professional allergy physicians, were collected at Peking Union Medical College Hospital from May to July 2019. The patients’ clinical information on age, gender, and allergic symptoms were collected. The basic information and clinical profiles of the participants in this study are shown in **Table 1**. These patients, from Beijing City and neighboring cities and provinces, had typical symptoms of allergic asthma (asthma), AR, and AD. The sIgE levels against *D. pteronyssinus* extract (D1) and *D. farinae* extract (D2) were measured using ImmunoCAP^®^ (Phadia, Sweden) (28), and all 548 samples were found positive. No pollen allergenic sources were involved.

**Table 1:** Demographics and characterization of the study population in the four age groups.

	Age group				Total	p-value
	2–12 years	13–18 years	19–45 years	46+ years		
Number	152 (27.7)	40 (7.3)	274 (50.0)	82 (15.0)	548 (100.0)	<0.001
Gender, male; number (%)	111 (73.0)	29 (72.5)	88 (32.1)	29 (35.4)	257 (46.9)	<0.001
Symptoms (%)						
Asthma	66 (43.4)	15 (37.5)	98 (35.8)	41 (50.0)	220 (40.1)	0.098
Allergic rhinitis	120 (78.9)	32 (80.0)	225 (82.1)	59 ( 72.0)	436 (79.6)	0.255
Allergic dermatitis	29 (19.1)	14 (35.0)	57 (20.8)	19 (23.2)	119 (21.7)	0.171
Specific IgE, median (IU/ml)						
Der p 1	3.06	1.03	0.63	1.09	0.97	0.0096
Der f 1	2.68	0.44	0.16	0.53	0.4	<0.0001
Der p 2	9.89	1.82	0.77	1.74	2.15	<0.0001
Der f 2	31.80	9.89	2.43	4.15	5.79	<0.0001
Der p 7	0.08	0.05	0.05	0.06	0.05	0.227
Der p 10	0.04	0.04	0.05	0.06	0.05	0.055
Der p 21	0.03	0.04	0.04	0.04	0.04	0.439
Der p 23	0.02	0.02	0.02	0.02	0.02	0.552

The allergic symptom evaluation was based on questionnaires, clinical observations, and tests. We defined three categories: AR in the nose and upper respirational tract; allergic asthma (AS) as a history of dyspnea, wheezing, and/or coughing episodes; and allergic skin disease including urticaria and dermatitis. The protocol of this study had the approval of the Ethics Committee of Peking Union Medical College Hospital (JS-3303). Written informed consent was obtained from all participants.

### 2.2 House dust mite-specific immunoglobulin E measurements

**Figure 1:**
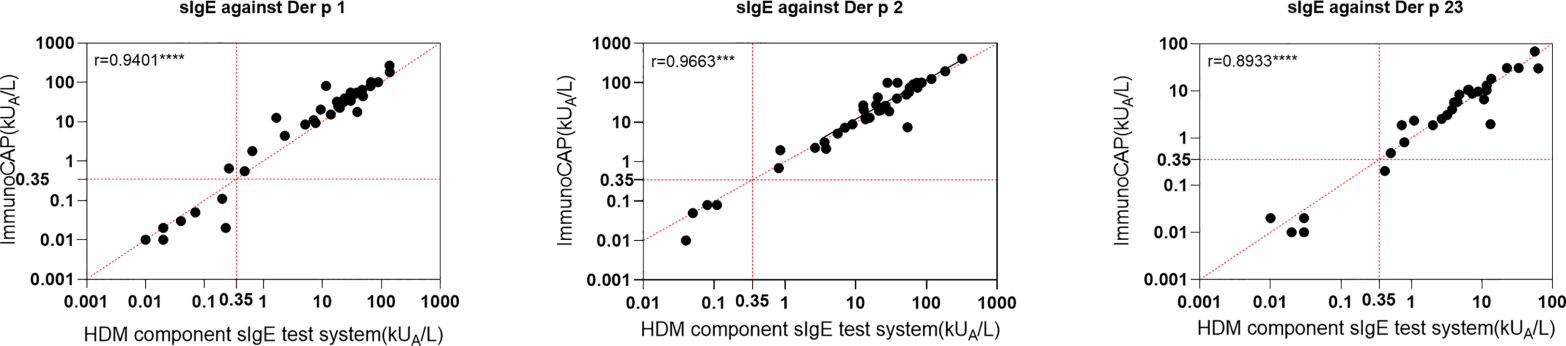
Linear regression between house dust mite component sIgE test system in this study and ImmunoCAP for three components. ***p < 0.001; ****p < 0.0001.

An HDM allergen component sIgE system from Hangzhou Zheda Dixun Biological Gene Engineering Co. Ltd. (hereinafter referred to as “HDM component sIgE test system”) was used to detect the sIgE level of serum samples and assess the risk of allergy. The HDM component sIgE test system uses a protein chip technology, and sIgE levels were determined with a DX-Autoblot 50 automatic immunoassay analyzer to reduce the measurement error. For serological testing, allergenic component proteins of *D. pteronyssinus* and *D. farinae* were used: Der p 1, Der p 2, Der p 7, Der p 10, Der p 21, and Der p 23 expressed in *E. coli*. Briefly, the coding sequences of corresponding allergens were cloned using specific primers and inserted into pET28a vector. After transformation into *E. coli* Rosetta (DE3), recombinant allergens were induced by 0.5 mM isopropyl-beta-D-thiogalactopyranoside at OD_600 = _0.6, and cells were grown at 16°C for 12 h. Then, cell pellets were harvested and disrupted by sonication. The soluble allergens with 6×His-tag were purified using Ni-NTA agarose (Sangon Biotech, China) following the manufacturer’s instruction, and Der f 1 and Der f 2 were purified from a *Pichia pastoris* culture by multi-step chromatography. In total, 250 μl of undiluted serum was applied to chips wetted using a washing buffer, diluted to working concentration, and incubated for 45 min. The secondary antibody was applied and incubated for 45 min after washing five times; then, the enzyme solution was added and incubated for 20 min after washing five times. Finally, the substrate was applied, and the chips were incubated for 20 min and washed five times. All steps were carried out using the DX-Autoblot 50 automatic immunoassay analyzer at 22°C. The built-in software (version 1.0) exported test results, and the serum samples with an antibody content of more than 0.35 IU/ml were considered positive according to a typical standard curve for sIgE determination (29). A previous research using this test kit has been published (12–14). The diagnostic performance of the HDM component sIgE test system was compared with three single components using ImmunoCAP for 39 patients’ sera. The agreement of positive and negative rates was 93%–100% in Der p 1, Der p 2, and Der p 23, and a significant linear correlation of sIgE values between the two assays was obtained (**Figure 1**).

### 2.3 Statistical analysis

Statistics Package for Social Science (SPSS version R27.0.0.0), an IBM statistical analysis software, and GraphPad Prism software (version 8.2.0, Fay Avenue, CA, USA) were used for statistical data analysis. The results were visualized with GraphPad Prism software.

Pearson chi-square or the Kruskal–Wallis test with 95% confidence intervals were used to compare two or more groups based on positive rates or sIgE levels. The Spearman correlation coefficient was used to assess the correlation between two groups, with a *p*-value less than 0.05 considered statistically significant.

## 3 Results

### 3.1 Patients’ characteristics

**Figure 2:**
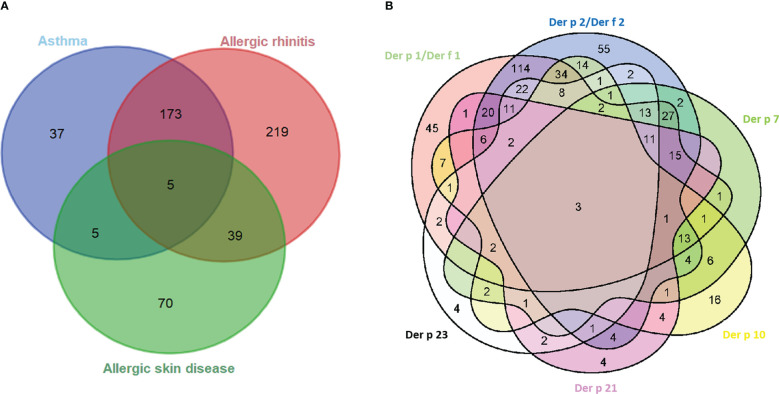
Venn diagrams of the interrelation of allergic symptom groups **(A)** and sensitized allergens **(B)**. Der p 1/Der f 1 and Der p 2/Der f 2 groups mean one or both of the components are positive.

Of the 548 HDM-allergic patients (**Table 1**), the average age was 27 years (range, 2–67 years), and 46.9% were male subjects. The majority had allergic rhinitis (79.6%), followed by allergic asthma (40.1%) and allergic skin disease (18.1%) (**Table 1**; **Figure 2A**). A component-resolved diagnosis demonstrated that Der p 1 (61.5%)/Der p 2 (52.4%) and Der f 1 (62.6%)/Der f 2 (69.5%) were the most frequently recognized allergen components in these patients from Northern China. Der p 10 (23.7%), Der p 7 (19.3%), Der p 21 (16.2%), and Der p 23 (16.6%) had a low positivity (**Figure 2**; **Table 2**).

**Table 2:** House dust mite component allergen sIgE-positive numbers and constituent ratio of three symptom groups.

Allergen	AS		AR		AD		p-value ^a^
	Number (%)	Medium	Number (%)	Medium	Number (%)	Medium	
Der p 1	133 (60.5)	0.87	271 (62.2)	1.08	75 (63.0)	0.85	0.875
Der f 1	114 (51.8)	0.40	233 (53.4)	0.45	54 (45.4)	0.19	0.296
Der p 2	137 (62.3)	2.76	286 (65.6)	2.81	64 (53.8)	0.52	0.060
Der f 2	151 (68.6)	7.46	316 (72.5)	8.84	72 (60.5)	2.65	0.040*
Der p 7	40 (18.2)	0.05	84 (19.3)	0.05	27 (22.7)	0.05	0.597
Der p 10	55 (25)	0.04	98 (22.5)	0.05	30 (25.2)	0.06	0.699
Der p 21	39 (17.7)	0.03	72 (16.5)	0.03	12 (10.1)	0.04	0.158
Der p 23	48 (21.8)	0.02	72 (16.5)	0.02	16 (13.4)	0.02	0.107

AS, allergic asthma; AR, allergic rhinitis; AD, allergic dermatitis.

a Comparing by chi-square test, the significant difference was based on the number of sIgE-positive samples. *p < 0.05.

### 3.2 HDM component-specific IgE detection refines the diagnosis of dust mite allergy

**Figure 3:**
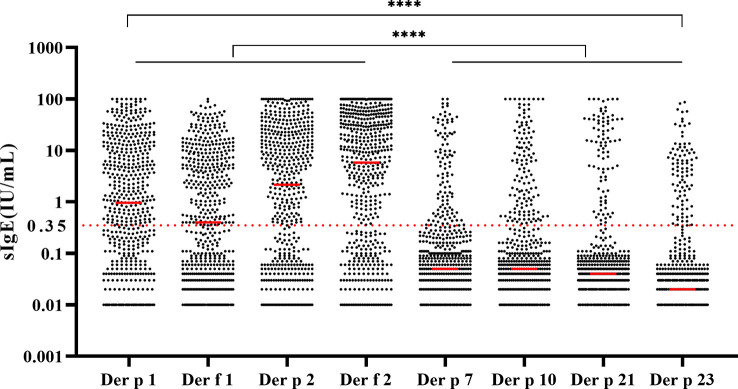
Levels and distribution of house dust mite component-specific IgE in 548 samples. All the groups were compared by Kruskal–Wallis test. ****p < 0.0001.

There were statistically significantly high sIgE levels of Der p 1, Der f 1, Der p 2, and Der f 2 compared with other components (**Figure 3**, *p* < 0.0001**)**. The sIgE levels against Der p 1 (*p* < 0.01), Der f 1 (*p* < 0.0001), Der p 2 (*p* < 0.0001), and Der f 2 (*p* < 0.0001) were significantly different between age groups (**Table 1**). However, the incidence of allergic clinical symptoms did not appear to be affected by age (**Table 1**).

**Figure 4:**
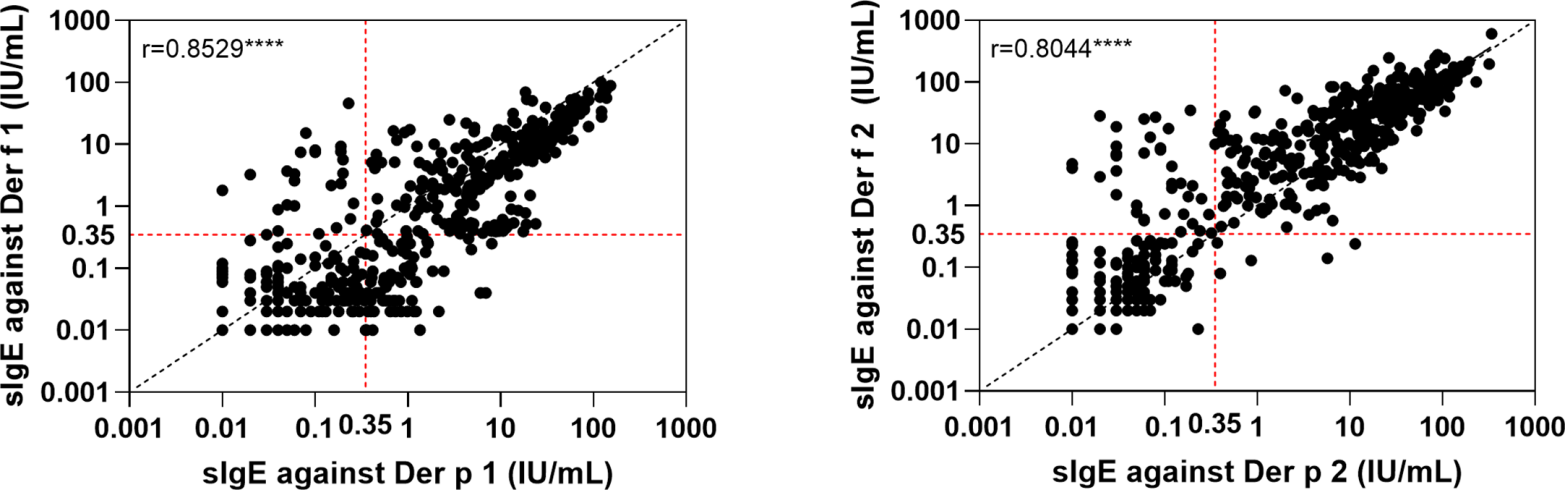
Linear regression of the two major components from two house dust mite species. ****p < 0.0001.

The relationships between the IgE levels of Der p 1 and Der f 1 were linear, as was that between Der p 2 and Der f 2 (**Figure 4**). We integrated the positive data from Der p 1 and Der f 1 as group 1 allergen (Der p 1/Der f 1) and from Der p 2 and Der f 2 as group 2 allergen (Der p 2/Der f 2) because of the similar biochemistry, and we investigated co-sensitization among the six HDM allergen groups of Der p 1/Der f 1, Der p 2/Der f 2, Der p 7, Der p 10, Der p 21, and Der p 23 (**Figure 2B**). This revealed that the highest co-sensitization level was in group 1 and group 2, with co-sensitization in a total of 302 patients (62.1%). Der p 7 was demonstrated and co-sensitive with other components, without any mono-sensitivity. Der p 10 was the third highest mono-sensitizer—3.3% of the subjects were responsive to Der p 10 alone, lower than group 2 (11.3%) and group 1 (9.3%). Der p 21 and Der p 23 had more co-sensitivity with group 1 and group 2 allergens.

### 3.3 Influence of age and gender in the detection of specific IgE against HDM components

**Figure 5:**
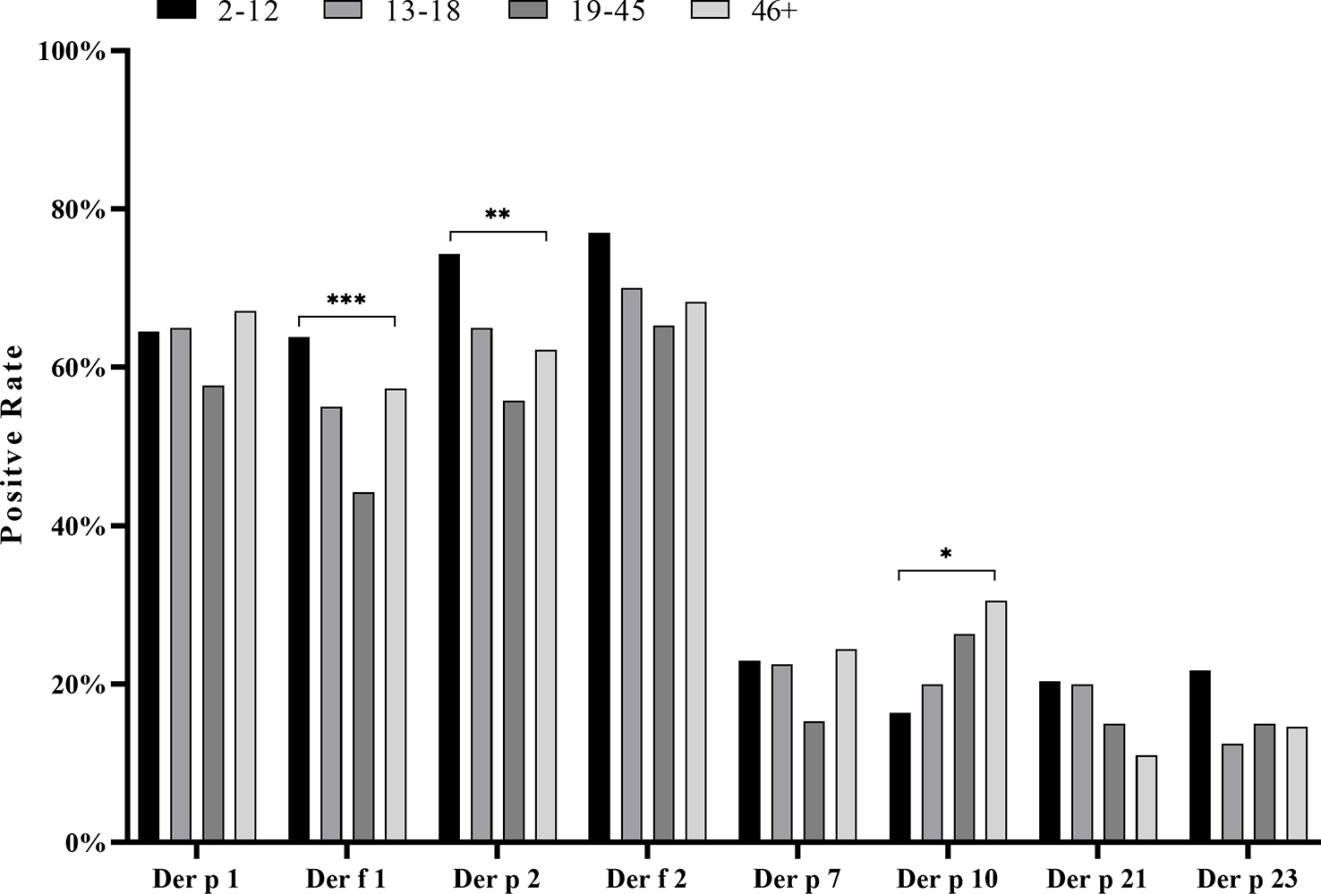
Positive rate of house dust mite component sIgE within four different age groups. Compared by chi-square test, the positive rate of each whole age group was analyzed. *p < 0.05; **p < 0.01; ***p < 0.001.

In our study, the positive value in female patients was slightly higher than that in males for most HDM component sIgEs, except for Der f 2 and Der p 10, but none of the disparities was statistically significant (*p >* 0.05). Analyzing the HDM component sIgE positive rates in different age groups, we found that children (2–12 years old), adolescents (13–18 years old), and the middle adult (46+ years old) had higher positive rates than the young adult age group (19–45 years) (**Figure 5**). Significant differences in positive rates of Der f 1 (*p* < 0.001), Der p 2 (*p* < 0.01), and Der p 10 (*p* < 0.05) were found between age groups. In addition, there was an increasing trend of a positive value of Der p 10 with age, while, in contrast, both Der f 1 and Der p 2 decreased with age at first and then rebounded after 46 years of age (**Table 1**; **Figure 5**).

### 3.4 The relationship between sIgE of HDM components and allergic symptoms

**Figure 6:**
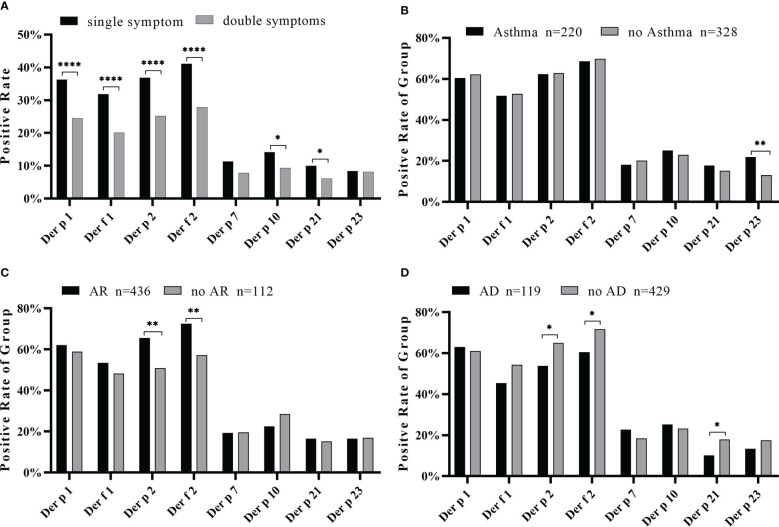
Positive rate of each component in simple or complex symptoms. **(A)** Positive rates of house dust mite component sIgE in one or two symptoms. The positive rate of component sIgE in the asthma group **(B)**, allergic rhinitis group **(C)**, and allergic skin disease group **(D)**. Compared by chi-square test, all data of positive rate of each group were analyzed. *p < 0.05; **p < 0.01; ****p < 0.0001.

In the relationship between the complexity of allergic symptoms and the allergenic components of HDM, we observed a strong sensitization of Der p 1, Der f 1, Der p 2, and Der f 2 among patients with a single symptom to multiple symptoms compared with other components (**Figure 6A**). The positive rate of the sIgE of most components was not significantly different between simple symptomatic and multi-symptomatic patients, except with Der p 23 (*p* < 0.05). The number of triple symptoms was five (**Figure 2A**), and this did not conform to statistical principles.

**Figure 7:**
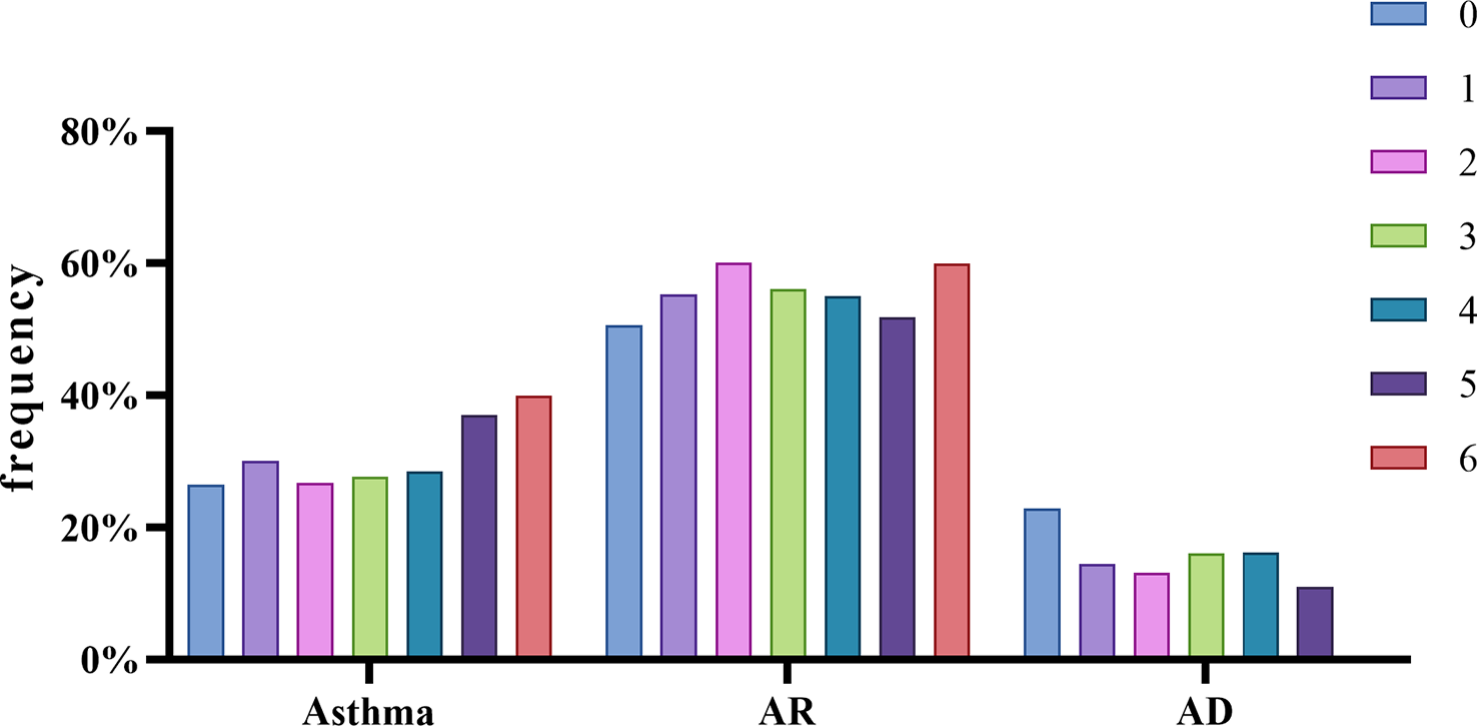
Different allergic phenotypes related to the number of sensitizing allergen component. Compared by chi-square test, all data of positive rate of each whole phenotype group were analyzed.

An analysis of the relationship between the symptoms and the positive frequency of HDM components revealed that Der p 23 was significantly higher in asthmatics (*p* < 0.01) (**Figure 6B**). Der p 2 and Der f 2 were significantly higher in patients with AR (**Figure 6C**) while lower in those with AD (**Figure 6D**). Moreover, the IgE levels of Der p 2 and Der f 2 were generally high in the AS and AR groups (**Table 2**). As for the impact of the number of positive components, there was an increasing trend in the frequency of asthma in line with the more sensitized allergens, while there was no such trend for allergic rhinitis and allergic skin disease (**Figure 7**).

## 4 Discussion

House dust mites are important allergen sources globally and can be seen almost everywhere in our lives: dust, curtains, bedding, carpets, sofas, towels, and even our food. Therefore, it is harder to avoid or eradicate than food and plant allergens. HDM causes allergic reactions such as allergic rhinitis, asthma, atopic dermatitis, and even severe anaphylaxis, which seriously affect the quality of life (30, 31). The current clinical diagnosis reliant on HDM extract can more accurately screen outpatients with HDM allergy, but it cannot provide more detailed and effective information for subsequent treatment. Nowadays, immune preparations using the allergen component protein of HDM have a more accurate treatment and better effect (32). Appropriate HDM component-specific antibody detection can clarify the molecular allergy map and help develop HDM component-targeted immune preparations. Considering that over 30 groups of HDM allergens have been identified, the six groups that we selected may not be sufficient, with 8% of the subjects not positive to any of the six group allergens tested. To improve the HDM molecular allergen test, more candidate allergens should be included for trial.

The subjects collected in our study were mainly from Northern China, where the prevalence of sIgE against Der p 1and Der p 2 was the lowest at approximately 40% (12). As shown in **Table 2**, the positive rates of Der p 1 and Der p 2 sIgE were 52.4% to 61.5% in the three symptom groups, similar to the 50%–65% positive rate published elsewhere (33) but lower than the data of Southern China (12, 15), possibly a regional feature related to temperature and humidity. The results showed that Der p 1, Der p 2, Der f 1, and Der f 2 in the sample had higher sIgE levels than the other components (**Table 1**), which might be determined by the characteristics of the component protein in content and sensitizing capacity (34).

The allergic characteristics of the HDM components in the three symptom groups are given in **Table 2** and **Figure 2**. A high overlap of Der p 1-, Der f 1-, Der p 2-, and Der f 2-positive subjects indicated that a considerable number of samples were positive because of these proteins being highly homologous as well as highly allergenic (35, 36). In the database of WHO/IUIS (http://www.allergen.org/viewallergen.php?aid=289; http://www.allergen.org/viewallergen.php?aid=274) and NCBI, Der p 1 and Der f 1 are both HDM cysteine proteases with 83% sequence homology and similar molecular weights, 24 and 27 kDa, respectively, with sensitization rates as high as 92% and 87%. Both Der p 2 and Der f 2 were 15-kDa NPC2 family protein with 87% sequence homology and sensitization rates of 71% and 90%, respectively. In addition, we also found that some samples were positive for an HDM component but negative for its highly homologous component. The reason may be amino acid sequence polymorphism (37), indicating that the allergic response to HDM is heterogeneous and diverse.

Der p 10 is a kind of tropomyosin, which has some homology with invertebrate tropomyosin (38). The positive rate of Der p 10 was low as shown in WHO/IUIS database (http://www.allergen.org/viewallergen.php?aid=290) but varied in different regions (5%–18%) (38). As a recognized cross-reactive HDM component protein, Der p 10 has been shown to have homology with the tropomyosin of shrimp and crab as a cross-reactive allergen (38). Generally, adults tend to have a more complex diet and consumption of more relevant seafood, so the increasing positivity in Der p 10 with age might be associated with this cross-reactivity. Patients sensitized to Der p 10 should also underwent the sIgE measurement of tropomyosin allergen in the relevant seafood, such as shrimp and crab, to check the possible food allergy, and then their diet is changed accordingly for safety. Der p 10 had a relatively higher mono-sensitization and seemed to play a role in the development of some symptoms in our study population.

The HDM allergic reaction is a complex physiological process involving the respiratory tract, mucous membrane, skin, and other tissues (39, 40). The pathogenesis of allergic diseases caused by distinctive allergens often involves IgE-mediated allergic reactions such as allergic rhinitis, allergic dermatitis, and allergic asthma (41).We attempted to explore the correlation between allergic symptoms and specific IgE antibodies of HDM components. The number of positive components were counted in each sample and grouped according to symptoms, and the result showed that the greater the number of positive components, the greater the probability of asthma (**Figure 7**). A significantly high positive rate of Der p 23 in the asthma group was observed (**Figure 6B**). Clinically related to asthma, Der p 23 sensitization had a high prevalence in HDM-sensitive people and was discovered in recent years (9, 22, 42). Walsemann and colleagues have shown that Der p 21 sIgE can affect the development of AD (43). In our study, the Der p 21 sIgE positive rate in the non-AD group was higher than in the AD group (**Figure 6D**), with the same trend for Der p 2 and Der f 2, indicating that Der p 21, Der p 2, and Der f 2 might play a more important role in respiratory allergic disease. In addition, the results of the comparative analysis showed that the positive rates of Der p 2 and Der f 2 increased in the AR group (**Figure 6C**), suggesting a possible association between AR and these allergens. Our results indicated that the HDM component detection system has distinct potential for helping with clinical diagnosis and enhancing the effects of treatment.

Gender and age are important factors that affect the AIT treatment of dust mite drops (44). However, in our study, the results for sIgE related to symptoms were not affected by gender, similar to Giorgio’s research (45).

In summary, using HDM component sIgE detection, we found the distribution characteristics of component sensitization in age and allergic diseases and the relationship between Der p 23, Der p 2, Der f 2, and Der p 21 and allergic symptoms. This provides more detailed molecular biological evidence for diagnosis and might technically solve the problem of universal treatment regardless of patient heterogeneity (46, 47). More research on the integration of routine CRD diagnosis and personalized treatment still needs to be done.

## Data availability statement

The original contributions presented in the study are included in the article/supplementary material. Further inquiries can be directed to the corresponding authors.

## Ethics statement

The studies involving human participants were reviewed and approved by the Ethics Committee of Peking Union Medical College Hospital. Written informed consent from the participants’ legal guardian/next of kin was not required to participate in this study in accordance with the national legislation and the institutional requirements.

## Author contributions

YL, LZ, and JW contributed equally to the work. All authors contributed to the article and approved the submitted version.
